# Dietary supplementation of nano-selenium eliminates the negative effects of long-term ivermectin injection on growth and reproductive performance of female rabbits

**DOI:** 10.5455/javar.2022.i577

**Published:** 2022-03-12

**Authors:** Set A. El-Shobokshy, Magda Ismail Abo-Samaha, Samia Mohamed Abd El-Rheem, Ferial Mohamed Sahwan, Gemechu Wirtu, Mosaad Abdel Khalek Soltan, Mohamed Emam

**Affiliations:** 1 Department of Nutrition and Veterinary Clinical Nutrition, Faculty of Veterinary Medicine, Alexandria University, Alexandria, Egypt; 2 Poultry Breeding and Production, Department of Animal Husbandry and Animal Wealth Development, Faculty of Veterinary Medicine, Alexandria University, Alexandria, Egypt; 3 Theriogenology Department, Faculty of Veterinary Medicine, Alexandria University, Alexandria, Egypt; 4 Animal Breeding and Production, Department of Animal Husbandry and Animal Wealth Development, Faculty of Veterinary Medicine, Alexandria University, Alexandria, Egypt; 5 Department of Biomedical Sciences, College of Veterinary Medicine, Tuskegee University, Tuskegee, AL, USA; 6 Department of Nutrition and Veterinary Clinical Nutrition, Damanhour University, Damanhour, Egypt

**Keywords:** Growth, ivermectin, nano-selenium, rabbit, reproduction

## Abstract

**Objective:**

We investigated the effects of a source of selenium [inorganic or nano-selenium (nano-Se)] on female V-line rabbits with or without injection of ivermectin (IVM).

**Material and Methods:**

Eighty four rabbits (12 weeks old) were randomly divided into 4 groups of 21 rabbits each with the basal diet supplemented as per the following treatments: G1 (control): inorganic Se at 0.3 mg/kg diet with no IVM injection; G2: inorganic Se with IVM injection; G3: nano-Se with no IVM injection; and G4: nano-Se with IVM injection. IVM was injected subcutaneously at 0.2 mg/kg body weight starting when the does were 14 weeks old and repeated weekly for five consecutive weeks.

**Results:**

Replacement of inorganic Se with nano-Se improved body weight and total body weight gain, total feed intake, average feed conversion ratio, and reproductive performance (age at puberty, number of service/conception, conception rate, number of kits/litter, and litter weight at birth). Similarly, sexual activity of does, serum estrogen levels, and serum levels of antioxidants (glutathione reduced, catalase, and malondialdehyde) increased in nano-Se-supplemented groups. Ivermectin treatment in inorganic Se-supplemented groups was detrimental to growth and reproductive performance, while these parameters improved in IVM-treated and nano-Se-supplemented groups.

**Conclusion:**

Nano-Se mitigated the negative effects of IVM treatment on the growth and reproductive performance of does.

## Introduction

The rabbit industry is growing fast in North African countries. There is also an increasing acceptance of rabbits as an animal protein source in the Mediterranean region [1,2]. Parasitic infestation, especially sarcoptic scabiei causing mange, is considered a major concern in commercial rabbit production [3]. Ivermectin (IVM) is usually used for the treatment and prevention of sarcoptic mange—a major disease with serious health consequences, including mortality in rabbits [4].

IVM is an acaricide and an anthelmintic drug derived from avermectin B1, which originates from *Streptomyces avermitilis*. When compared to treatments via oral and topical routes, subcutaneous administration of IVM provides better bioavailability [5]. The drug is generally well tolerated in mammals [6,7]. However, repeated doses of IVM caused pathological changes in hepatic tissue, such as vacuolation of hepatocytes and fibrosis in female rabbits [8]. In addition to various pathological changes in male rats’ liver, kidneys, and testis, therapeutic and double therapeutic doses decreased total sperm count and caused mortalities. Some pathological changes include congestion of blood vessels, degenerative changes such as vacuolar, hydropic, or even necrotic changes, and functional disorders in the liver and kidneys [9]. The usual prophylactic treatment regimen for IVM practiced by rabbit breeders is to inject IVM every 2 weeks.

With the advent of nanotechnology, nanoparticles embedded in trace mineral supplements have many beneficial properties different from those of inorganic minerals [10]. Selenium (Se) is essential for animals, as it aids in growth, fertility, and endocrine and metabolic processes in a variety of organs [10,11]. Although the supplementation of selenium has become common for different species, emerging data indicate that selenium nanoparticles have high bioavailability, catalytic efficiency, and absorbability, but low toxicity compared with other selenium sources [12]. Se has been shown to help different types of animals, such as ewes [14] and buffaloes [15], improve their redox and health status, as well as their productivity and reproductive performance [16,17].

Antioxidants protect against the damaging effects of free radicals originating from normal metabolism [18]. The antioxidant activity of Se is exerted by increasing glutathione reduced (GSH)-Px activity [19] and enhancing the immune system [18]. It also reduces lipid peroxides and hydrogen peroxide and improves lipid oxidative stability, thus preserving meat quality [20]. Supplementation of the diet with nano-Se, when compared with the control group, enhanced final body weight, feed conversion ratio (FCR), and daily body weight gain in heat-stressed rabbits without affecting feed consumption [16].

IVM treatment is vital in controlling parasitic infections in different animal production systems, including rabbits. However, its adverse effects have been a major concern. Kumar et al. [21] mentioned that adding vitamins to IVM improves the parasitological and clinical recovery in rabbits infected with sarcoptes scabiei compared to that of IVM alone. Providing Se in the diet helps restore the imbalance between oxidants and antioxidants by providing more antioxidants. The aim of this experiment was to detect the effects of the source of Se (inorganic Se and nano-Se) with ivermectin treatment on the growth and reproductive performance of does. So, we looked into whether selenium in the diet could help lessen some of the IVM treatment effects.

## Material and methods

### Ethical approval

The experimental procedure applied in this study was approved by Alexandria University‘s Institutional Animal Care and Use Committee (Permit #2021/013/96). 

### Experimental design, feeding program, and animal management

Rabbits were housed under the same conditions during the whole experimental period. This experiment used 84 female V-line rabbits (12 weeks old). The V-line is a line (hybrid of New Zealand White and California Breed) that was developed in 1982 at the Department of Animal Science at the Universidad Politecnica de Valencia in Spain [22] and was imported to Alexandria University. Animals were divided into four groups (21 does/group), and each group was divided into three replicates (seven does/replicate). The does were fed an isonitrogenous and isoenergetic basal diet containing all the required nutrients [23], except for selenium [24,25]. Table 1 shows the ingredients of the basal diet. The treatment groups were Group 1 (G1) does were fed on the basal diet supplemented with inorganic Se at 0.3 mg/kg diet (0.657 gm selenium selenite per kg mineral premix), without IVM injection (control group); Group 2 (G2) does were fed the basal diet supplemented with inorganic Se with IVM injection; Group 3 (G3) does were fed on the basal diet with replacement of inorganic Se by nano-Se without IVM injection; and Group 4 (G4) does were fed on the basal diet with replacement of inorganic Se by nano Se, with IVM injection.

Nano-Se was obtained from NanoShell, Wilmington, Delaware. IVM (Alfasan International B.V. Company, The Netherlands) was administered by S/C injection at a dose of 0.2 mg/kg body weight. The IVM injection was initiated when the does were 14 weeks old (2 weeks after the beginning of the experiment) and repeated weekly for five consecutive weeks until 18 weeks of age. A pelleted diet was provided *ad libitum* for the animals during the whole experiment. Individual rabbits were housed in galvanized wire net batteries with an automatic drinker and a manual feeder (width* length* height: 44 cm*50 cm*35 cm). The rabbits were raised in an open house system with a 16-h light and an 8-h dark cycle (naturally ventilated room with windows and ceiling fans). Fresh tap water was provided at all times through stainless steel nipples placed inside each cage.

### Growth performance parameters

The does were weighted at the beginning (12 weeks) and then for every 2 weeks till the 20th week of age. The final and initial body weight difference was used to compute the total gain. Feed intake was recorded; the average feed conversion ratio (AFCR) was calculated as kg of total feed intake (TFI)/kg of total gain.

**Table 1.** The components and chemical composition of basal diet experimental ration.

Ingredients	%
Corn	5
Barley	15
Wheat bran	25
Oil	3
Molasses	3
B Hay	32.2
SBM 42.9	14.7
Meth	0.2
Limestone	0.2
MCP	0.9
Salt	0.5
Vitamin premix^1^	0.15
Mineral premix^2^	0.1
Anti-mycotoxin	0.05
Total	100
**Calculated analyses (NRC**[23])	
Digestible energy (kcal/kg)	2,588.5
Crude protein (%)	17.05
Ether extract (%)	5
Crude fiber (%)	11.97
Starch	15.7

1 1 kg of vitamin premix contained vitamin A (10,000 IU), vitamin D3 (1,800 UI), vitamin E (15 mg); vitamin K3 (4.5 mg), vitamin B1 (0.5 mg), vitamin B2 (4 mg), vitamin B12 (0.001 mg), folic acid (0.1 mg), pantothenic acid (7 mg), and nicotinic acid (20 mg).

2 The components of each kilogram of mineral premix are Mn sulfate (34.55 gm), Zn oxide (74.63 gm), iron carbonate (207.47 gm), copper oxide (12.52 gm), cobalt oxide (0.42 gm), K iodide (0.26 gm), Se selenite (0.657 gm), and carrier (limestone) up to 1 kg. Minerals provided per kilogram of diet are manganese (8.5 mg), zinc (60 mg), iron (100 mg); copper (k10 mg), cobalt (0.3 mg), iodine (0.2 mg), and Se (0.3 mg).

### Sample collection

At 20 weeks of age, 9 does from each group (3 from each replicate) were chosen at random for blood sampling that was collected from the marginal ear vein in non-heparinized sterile tubes. Blood samples were kept at room temperature for 30 min, then centrifuged for 15 minutes at 4,000 rpm, and serum samples were stored at −20°C for further analysis of selenium (Se), glutathione reduced (GSH), catalase (CAT), malondialdehyde (MDA), and estrogen. Serum Se, GSH, and CAT were assessed according to the manufacturer’s instructions by spectrophotometric procedures (Hitachi spectrophotometer, Tokyo, Japan) using commercially available kits (Biodiagnostic Co., Dokki, Giza, Egypt). Serum MDA reported as nm/ml was determined by the colorimetric method, which relies on the reaction between MDA and thiobarbituric acid [26]. The obtained optical density results were expressed as nmol/ml. The serum concentrations of estrogen were determined using the mouse estrogen ELISA kit (B9 Bld, High-Tech Medical Devices Park, Wuhan, Hubei, China).

### Reproductive performance

After reaching the puberty in all groups, each doe was individually mated by transferring the female to a cage with a mature male. Each doe was returned to its treatment cage immediately after mating. The doe’s sexual receptivity was assessed by examining her behavior with a male. If spontaneous mating did not occur within 5 min, then it was considered unwilling to mate. Unwilling to mate includes aggression against the male, moving to a corner of the cage, and circling within the cage, whereas willing to mate included the doe permits mounting but no lordosis posture and allows mounting with a lordosis posture. The number of services per conception was evaluated. When does failed to conceive, they were carefully inspected for signs of receptivity before being returned to the buck for further mating. On the 15th day after mating, abdominal palpation was carried out to examine the pregnancy. The number of kits/litters and weight were recorded at birth.

### Statistical analysis

The SAS software one-way analysis of variance feature was used to analyze the data [27]. *Post-hoc* analysis was carried out using Duncan’s test. The results were expressed as mean standard error, with *p* < 0.05 set as the overall significance level.

## Results

### Growth performance

The influence of different selenium sources in the diet with or without IVM injection on the growth performance of doe rabbits is shown in Table 2. The replacement of inorganic Se by nano-Se in Group 3 significantly (*p* < 0.0001) increased the BW by about 15.0%, 12.5%, 8.0%, and 11.0% at the 14th, 16th, 18th, and 20th week of age, respectively, compared with the control group fed on inorganic Se (G1). Similarly, does in G3 fed on nano-Se had a significantly (*p *< 0.0001) higher total body weight gain (TBWG), TFI, and AFCR by about 30.5%, 13.4%, and 18.8%, respectively, when compared with the G1 fed on inorganic Se.

Compared to G1 (control) and G2 animals receiving IVM treatment and inorganic Se supplementation, BW decreased by about 3.9% (*p *> 0.05), 6.3% (*p* < 0.0001), and 7.5% (*p* < 0.0001) at the 16th, 18th, and 20th week of age, respectively, demonstrating the detrimental effect of IVM treatment even when supplementing the diet with inorganic Se. Similarly, TBWG and TFI declined significantly (*p* < 0.0001) by about 20.2% and 17.4%, respectively, in G2, injected with IVM compared with G1.

**Table 2.** Influence of dietary inorganic or nano-selenium supplementation with or without ivermectin treatment on growth performance of rabbits does.

Variable	Group				*p*-value
	G1	G2	G3	G4	
Initial body weight at 12 wks (kg)	1.632 ± 0.009	1.638 ± 0.007	1.618 ± 0.025	1.612 ± 0.017	NS
Body weight at 14 wks (kg)	1.798 ± 0.048^b^	1.770 ± 0.014^b^	2.068 ± 0.033^a^	1.998 ± 0.027^a^	<0.0001
Body weight at 16 wks (kg)	2.120 ± 0.041^c^	2.037 ± 0.018^c^	2.385 ± 0.031^a^	2.278 ± 0.036^b^	<0.0001
Body weight at 18 wks (kg)	2.422 ± 0.041^b^	2.270 ± 0.022^c^	2.615 ± 0.020^a^	2.555 ± 0.039^b^	<0.0001
Final Body weight at 20 wks (kg)	2.632 ± 0.059^c^	2.435 ± 0.018^d^	2.922 ± 0.030^a^	2.752 ± 0.030^b^	<0.0001
TG (kg)	1.000 ± 0.065^c^	0.798 ± 0.019^d^	1.305 ± 0.015^a^	1.140 ± 0.022^b^	<0.0001
TFI (kg)	5.518 ± 0.100^b^	4.550 ± 0.172^c^	6.250 ± 0.020^a^	6.325 ± 0.001^a^	<0.0001
AFCR	5.885 ± 0.276^a^	5.703 ± 0.153^a^	4.792 ± 0.039^b^	5.588 ± 0.104^a^	<0.0001

The results are displayed as mean ± standard error. Within the same row, means with various superscripts differ significantly (*p *< 0.05). NS: non-significant, TG: total gain, TFI: total feed intake, and AFCR: average feed conversion ratio.

**Figure 1.**
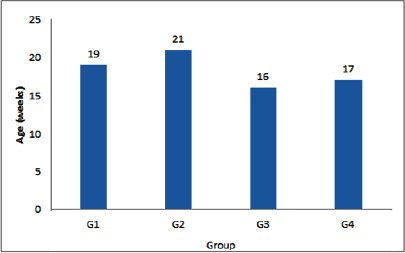
Effects of dietary inorganic or nano-selenium supplementation with or without ivermectin treatment at the age of puberty on rabbit does. G1, control: inorganic Se at 0.3 mg/kg diet and no IVM injection; G2: inorganic Se and IVM injection; G3: nano-Se and no IVM injection; and G4: nano-Se with IVM injection. IVM was injected subcutaneously at 0.2 mg/kg body weight starting when does were 14 weeks old and repeated weekly for five consecutive weeks.

Replacement of inorganic Se by nano-Se and IVM treatment (G4) eliminated the negative impacts of IVM on the growth of does and significantly (*p < *0.0001) enhanced the BW at the 16th, 18th, and 20th week of age by about 12.9%, 11.8%, 12.5%, and 13.0%, respectively, and TBWG and TFI by about 42.9% and 39.0%, respectively. It also numerically but non-significantly improved AFCR throughout the experiment by about 2.0 compared with G2 injected with IVM and fed with inorganic Se.

### Reproductive performance

Age at puberty (Fig. 1) differed significantly among groups (*p* < 0.0001), where G3 reached puberty at the age of 16 wks, followed by G4 (17 wks), G1 (19 wks), and G2 (21 wks). The results of the reproduction are presented in Table 3. The number of services per conception was lowest in G3 and G4, while the highest value was recorded in G2, with a significant difference among different groups. On the other hand, the conception rate was highest in G3 and G4 but lowest in G2. The number of kits/litter was significantly different among groups, where G3 and G4 were about the same value, followed by G1 (control) and G2. Litter weight was significantly different among groups, with >10 gm difference between G4 and G1 (control) in litter weight at birth.

**Table 3.** Effects of dietary inorganic or nano-selenium supplementation with or without ivermectin treatment on the reproductive performance of rabbit does.

Variable	Group				*p*-value
	G1	G2	G3	G4	
Number of service/conception	2.00 ± 0.14^b^	2.57 ± 0.18^a^	1.19 ± 0.09^c^	1.24 ± 0.10^c^	<0.0001
Conception rate	33.20 ± 0.058^c^	11.37 ± 0.265^d^	77.76 ± 0.006^a^	44.43 ± 0.015^b^	<0.0001
Number of kits/litter	7.00 ± 0.00^b^	5.14 ± 0.17^c^	7.57 ± 0.11^a^	7.24 ± 0.17^ab^	<0.0001
Litter weight at birth (gm)	48.78 ± 0.07^d^	53.02 ± 1.49^c^	59.62 ± 0.27^a^	56.19 ± 0.90^b^	<0.0001

The results are displayed as mean ± standard error. Within the same row, means with various superscripts differ significantly (*p *< 0.05).

**Figure 2.**
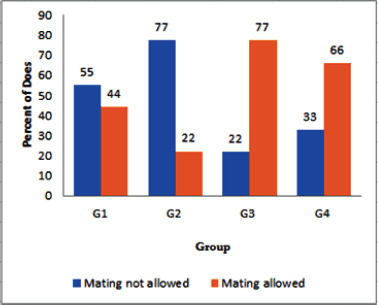
Effects of dietary inorganic or nano-selenium supplementation with or without ivermectin treatment on the sexual activity of rabbit does. G1, control: inorganic Se at 0.3 mg/kg diet and no IVM injection; G2: inorganic Se and IVM injection; G3: nano-Se and no IVM injection; and G4: nano-Se with IVM injection; IVM was injected subcutaneously at 0.2 mg/kg body weight starting when does were 14 weeks old and repeated weekly for five consecutive weeks.

The sexual activity of female rabbits is shown in Figure 2. The percentage of females that did not allow mounting was highest in G2, followed by G1 and G4, and lowest in G3. The percentage of females allowing mounting was also significantly different among groups, with the highest percentage observed in G3, followed by G4, G1, and then G2. Serum estrogen levels of female rabbits are presented in Table 4. Estrogen hormone levels varied significantly across groups, with the highest in G3 and G4, followed by G1 and G2. 

### Antioxidant indicators

Means and standard errors for the effect of different treatments on the concentration of antioxidant enzymes, e.g., GSH and CAT, and Se levels, are presented in Table 4. The nano-Se non-injected results indicated that nano-Se supplementation in the basal diet of IVM injected or non-injected doe was associated with considerably elevated antioxidant activity. Supplemented nano-Se significantly (*p* < 0.0001) increased the serum Se level to 1.65 gm/ml in the female group that did not receive IVM injection. On the other hand, the inorganic Se-injected group showed the least Se level in serum (0.61 µgm/ml; *p* < 0.0001). Se content was significantly higher (1.33 µgm/ml; *p* < 0.0001) in the nano-Se-injected group compared to 0.91 and 0.61 µgm/ml for the inorganic Se non-injected and injected groups, respectively. Regarding the GSH enzyme, higher serum levels were reported for nano-Se-injected and non-injected groups. The mean of serum GSH was 49.3 and 49.0 mg/dl for the nano-Se-injected and non-injected groups, respectively. On the other hand, a significantly lower GSH level (23.33 mg/dl, *p* < 0.0001) was observed in the inorganic Se-injected group. Similarly, female rabbits in the nano-Se non-injected group showed higher serum CAT (60.00 U/ml, *p* < 0.0001), and it was followed by the nano-Se-injected group (51.00 U/ml, *p* < 0.0001). On the other hand, lower serum CAT was recorded for the inorganic Se IVM-injected group (20.67 U/ml, *p* < 0.0001). 

In comparing the non-injected groups (supplemented nano-Se *vs*. inorganic Se), the present study demonstrated that serum Se, GSH, and CAT levels were significantly higher in the nano-Se non-injected group compared to the inorganic Se non-injected group. Table 4 shows the effect of dietary nano-selenium on MDA (as an index of the oxidative process) in relation to the MDA enzyme. Lower serum MDA was reported for the nano-Se non-injected group (3.60 nmol/ml).

The means of serum MDA were 3.60, 4.37, and 4.47 nmol/ml (*p* > 0.05) in the nano-Se non-injected group, inorganic Se non-injected group, and nano-Se-injected group, respectively. On the other hand, higher serum MDA was recorded for the inorganic Se-injected group (11.83, *p* < 0.0001).

## Discussion

Se is a crucial element that regulates the body’s energy, protein anabolism, and oxidative stress, allowing animals to develop and reproduce normally [28]. However, recent research reveals that the NRC Se standard of 0.08 mg/kg in a developing rabbit diet failed to meet the rabbit’s growth requirements and that high levels are required to optimize nutrient digestion, absorption, and utilization, which boosts productive and reproductive performance [13,24,28].

We found that feeding female rabbits on nano-Se instead of inorganic Se significantly improved the live BW, TBWG, TFI, and AFCR throughout the experimental period led to the highest gain and feed intake and, therefore, the best growth performance and feed conversion. Similarly, Dawood et al. [29] indicated that feeding dietary nano-Se to Nile tilapia boosted final BW, weight gain, and growth rate, as well as improved FCR. In line with the same trend, Emara et al. [30] and Abdel-Wareth et al. [16] reported an improvement in the growth rate, BW, and FCR of rabbits fed on nano-Se and explained this by improving the nutrient digestibility, antioxidative, and immune properties and increasing the serum active thyroid hormone level [31]. Similarly, Tag-El Din [24] reported that feeding nano-Se (0.3 mg/kg diet) showed the best growth performance and FCR of growing New Zealand White rabbits and attributed this to the high biological activity and immune regulation of nano-Se [32]. Mohapatra et al. [10] indicated that pullets or broilers’ diets supplemented with 0.3 mg/kg nano-Se led to the best growth performance. Moreover, Mohamed et al. [33] mentioned that nano-Se improved the feed intake of doe rabbits. Similarly, Marounek et al. [34] also indicated that Se supplementation improved BWG, FI, and FCR of growing rabbits as antioxidant properties prevented the damaging effect of free radicals and pathogens. It also protected the intestinal mucosa and improved its absorptive capacity, preventing diarrhea by decreasing the peristastic movement of the intestine [35]. In contrast to our results, of Abdel-Wareth et al. [16], Tag-El Din [24], and El-Kazaz et al. [36] revealed that nano-Se reduced the TFI in growing NZW rabbits, male Californian rabbits, and Japanese quails, respectively. Such variations may be attributed to using different doses, sex, or species.

IVM is an effective drug for treating and preventing parasitic infections [37]. However, the continuous use of IVM has a cytotoxic effect and induces DNA damage, apoptosis, and oxidative degradation [38]. Our results showed that the repeated IVM injections significantly deteriorated the growth performance and feed intake with dietary inorganic Se. Interestingly, the replacement of inorganic Se by nano-Se eliminated the detrimental effects of IVM on growth performance and FI. Similar to our findings, Chahrazed et al. [39] used rabbits to evaluate the adverse effects of an elevated dose of IVM (2 mg/kg BW S/C, 3 doses per week) for three consecutive weeks and concluded that the high dose of IVM significantly (*p* < 0.05) lowered the TFI because of the decreased appetite, which then decreased total gain and final rabbit live BW. Chahrazed et al. [39] concluded that adding vitamin C to IVM protects against lowered body weight, gain, and feed consumption. A study in male Wistar albino rats showed that selenium could help male rats recover from IVM-induced toxicities [40]. 

**Table 4.** Effects of dietary inorganic or nano-selenium supplementation with or without ivermectin treatment on serum concentration of estrogen, Se, GSH, CAT, and MDA in rabbit does at 20th week of age.

Variable	Group				*p*-value
	G1	G2	G3	G4	
Estrogen (pg/ml)	43.67 ± 2.33^b^	21.00 ± 0.87^c^	71.00 ± 1.15^a^	70.33 ± 3.06^a^	<0.0001
Selenium (µg/ml)	0.91 ± 0.06^c^	0.61 ± 0.02^d^	1.65 ± 0.09^a^	1.33 ± 0.04^b^	<0.0001
GSH (mg/dl)	40.67 ± 1.92^b^	23.33 ± 1.17^c^	49.00 ± 1.15^a^	49.33 ± 2.52^a^	<0.0001
CAT (U/ml)	43.67 ± 1.88^c^	20.67 ± 0.88^d^	60.00 ± 0.58^a^	51.00 ± 0.76^b^	<0.0001
MDA (nmol/ml)	4.37 ± 0.19^b^	11.83 ± 0.70^a^	3.60 ± 0.12^b^	4.47 ± 0.20^b^	<0.0001

The results are displayed as mean ± standard error. Within the same row, means with various superscripts differ significantly (*p *< 0.05). GSH: glutathione and MDA: malondialdehyde.

Rabbit producers focus on a high conception rate, high receptivity, large litter size, and little or no mortality for successful rabbit production [41,42]. Many researchers explain how Se affects reproduction and why it is necessary for reproductive tissues [43,44]. Antioxidants stimulate the process of steroid genesis and stimulate the anterior pituitary gland to release GnRH hormones and initiate folliculogenesis in the ovaries [43]. Thus, Se could improve uterine health by enhancing neutrophil function, supporting uterine function, and stimulating ovarian activity [45]. This may explain our results that the group supplemented with nano-Se had the best age of puberty, conception rate, number of kits/litter, litter weight, and lower number of services per conception, which is associated with higher values of conception rate. Azoz and El-Kholy [46] reported similar results in matings per conception. The fact that the IVM-injected group had the worst reproductive parameters raises doubts about the safety of IVM as an antiparasitic treatment for animals. The balance between oxidants and antioxidants may be disrupted by IVM [47] by producing cytotoxic free radicals, such as nitric oxide, which is also the mechanism by which IVM exerts its antiparasitic effects [48].

Oxidative stress has been linked to infertility, implantation failures, and pregnancy loss [49,50]. Reactive oxygen species affect a lot of reproductive processes, including folliculogenesis, oocyte maturation, ovulation, corpus luteum formation, and regression, as well as implantation and fetal development [51]. Al-Hizab and Mostafa [50] reported that doramectin administration induced a number of pathological changes, including congestion of ovarian blood vessels and endometrial edema after 3 weeks of double therapeutic dose and ovarian degeneration, leading to excessive atresia of follicles after a prolonged period of injection of the drug. Similar observations were also made in Guinea pigs after double and triple therapeutic doses of doramectin injection. Besides cytotoxic effects, the free radicals resulting from the drug treatment disrupt many steps in the production of hormones essential for embryonic development [52].

Estrogen is the primary reproductive hormone that affects the reproductive tract‘s growth, development, maturation, and function. It is also involved sexual differentiation and behavior [53]. In our study, the deleterious effect of IVM on female reproduction was associated with a lower serum estrogen level in G2 as compared to control. This is likely due to the IVM-induced shift of steroidogenesis from estrogen to testosterone [54] and cortisol [55]. These shifts aggravate disruptions in normal reproductive processes and suggest increased stress levels in treated animals. In our study, nano-Se showed improvement in estrogen levels in G3 and G4 as compared to control. This indicates that nano-Se helped overcome the effect of IVM in G4 by improving the level of estrogen and demonstrating the vital role selenium plays in mitigating oxidative damage [44]. 

Our results showed that nano-Se had pronounced antioxidant activity by significantly improving the enzymatic activities of Se, GSH, and CAT. Our findings are consistent with previous reports that dietary Nano-Se improved GSH activity in the serum of poultry [17,56] and rabbits [19,56]. Moreover, the improvement in CAT was also reported in nano-Se-supplemented rabbits [57]. 

Lipid oxidation is a severe concern in rabbit meat due to the large amount of polyunsaturated fatty acids, which are sensitive to oxidation, reducing meat quality, and shortening meat shelf life due to rancidity and color deterioration [42]. MDA is a polyunsaturated fatty acid metabolite that is used as a marker of oxidative stress. It was clear from our finding that IVM injection adversely affected the immune status of inorganic Se-supplemented rabbits, as observed by the decreased antioxidant enzymatic activity and increased MDA levels (a marker of oxidative stress). GabAllh et al. [52] also stated that the injection of the therapeutic dose of IVM in female rabbits resulted in lymphoid depletion in the white pulp of the spleen with thickening in the splenic capsule. The harmful and stressful effects of repeated IVM administration in female rabbits were also reported by Al-Jassim et al. [8]. Our study demonstrated that the inclusion of nano-Se in the rabbit diet, regardless of IVM treatment, reduced MDA levels. Therefore, our results highlighted the important role of nano-Se in alleviating the harmful and stressful effects caused by IVM injection and improving the lipid stability of rabbit meat. This result is due to the increased selenium concentration caused by nano-Se supplementation, as well as its protective role through immunological activity [58], which boosts both cellular and humoral immune responses [59]. Nano-Se supplementation also minimizes the negative effects of oxidative stress on the liver and stimulates immunoglobulin synthesis [60]. 

Previous investigations have shown that selenium can improve lipid stability [61,62]. Sheiha et al. [57] observed that nano-Se treatment significantly reduced MDA production in heat-stressed rabbits when compared to non-heat-stressed ones. It was also reported that rabbits treated with nano-Se had lower serum MDA levels [56].

According to our findings, dietary nano-Se supplementation resulted in considerable antioxidant activity in comparison to inorganic selenium. In comparison to inorganic selenium, Nano-Se is more effective and efficient at upregulating selenoenzymes [56]. Furthermore, nanoparticles’ greater performance can be attributed to their small particle size and large surface area, increased mucosal permeability, improved intestinal absorption, and tissue deposition [63].

## Conclusion

Replacement of inorganic Se by nano-Se enhanced the body weight, weight gain, and FCR in female rabbits, as well as the reproductive performance. Supplementation of nano-Se in the diet of rabbits injected with ivermectin eliminated the negative effects of ivermectin and improved the performance of female rabbits. We recommend including nano-Se in the diets of rabbits exposed to ivermectin injection. 

## List of abbreviations

AFCR, Average feed conversion ratio; BW, Body weight; CAT, Catalase; FCR, Feed conversion ratio; FI, Feed intake, GSH, Glutathione reduced; G, Group; IVM, Ivermectin; MDA, Malondialdehyde; Se, Selenium; TBWG, Total body weight gain; TFI, Total feed intake.
